# Supplementary material for: Human C15orf39 Inhibits Inflammatory Response via PRMT2 in Human Microglial HMC3 Cell Line
Source: Int J Mol Sci. 2024 May 30;25(11):6025. doi: 10.3390/ijms25116025 (PMC11173073; doi:10.3390/ijms25116025)
Supplement: Supplementary file 1 [file ijms-25-06025-s001.zip › ijms-3022831-supplementary.pdf]

International Journal of *Molecular Sciences*

MDPI

Supplementary Materials

Human C15orf39 Inhibits Inflammatory Response via PRMT2 in Human Microglial HMC3 Cell Line

Min Zhang [1,2], Yaqi Xu [1,2], Gaizhi Zhu [1,2], Qi Zeng [1,2], Ran Gao [1,2], Jinming Qiu [1,2], Wenting Su [1,2,*] and Renxi Wang [1,2,*]

1 Beijing Institute of Brain Disorders, Laboratory of Brain Disorders, Ministry of Science and Technology, Collaborative Innovation Center for Brain Disorders, Capital Medical University, Beijing 100069, China
2 Laboratory for Clinical Medicine, Capital Medical University, Beijing 100069, China
* Correspondence: renxi_wang@ccmu.edu.cn; wtsu@ccmu.edu.cn

Supplementary Materials

Supplementary Table S1. The primers for C15orf39, IL-6, TNFα, and ACTIN.

**human C15orf39-F**	**5′-GGCAGCTAGGAGGGTTGCT-3′**
human C15orf39-R	5′-CTCCTAGGCCCCTGCTACTC-3′
human IL-6-F	5′-GTACATCCTCGACGGCATCTC-3′
human IL-6-R	5′-GCTCTGGCTTGTTCCTCACTAC-3′
human TNFα-F	5′-TGTAGCCCATGTTGTAGCAAACC-3′
human TNFα-R	5′-GAGGACCTGGGAGTAGATGAGGTA-3′
human ACTIN-F	5′-TCCCTGGAGAAGAGCTACGA-3′
human ACTIN-R	5′-ATCTGCTGGAAGGTGGACAG-3′

F: forward primers; R: reverse primers.

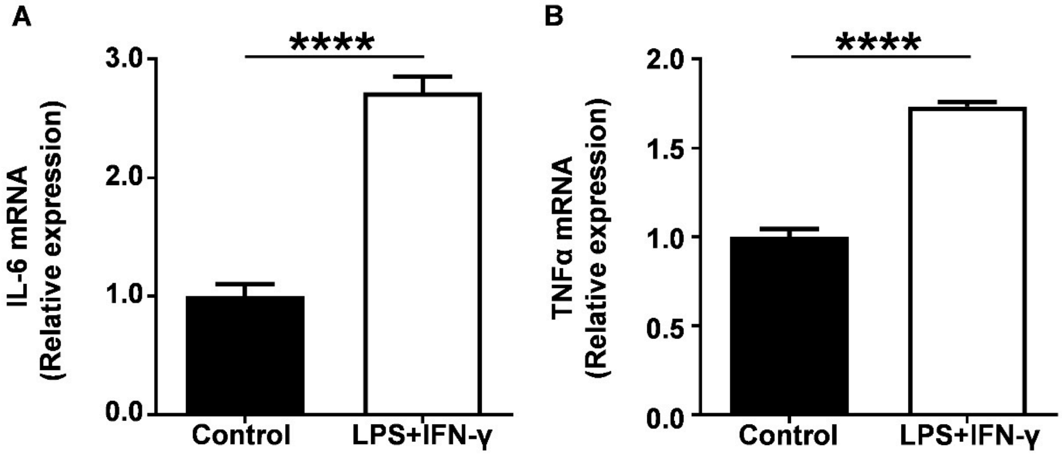

Supplementary Figure S1. LPS/IFN-γ induced IL-6 and TNFα expression in in the human microglial clone 3 cell line (HMC3). HMC3 cells were stimulated by LPS/IFN-γ for 24 h and collected. IL-6 (A) and TNFα (B) mRNA expression was determined by qPCR assay. (**A**, **B**) Data indicate means ± standard deviation (SD), N = 3, two tailed Student's t-test, ****$p < 0.0001$.
